# Case Series: Cyclops lesion - extension loss after ACL reconstruction

**DOI:** 10.4103/0971-3026.69361

**Published:** 2010-08

**Authors:** Sunita Dhanda, Darshana Sanghvi, Dinshaw Pardiwala

**Affiliations:** Department of Diagnostic and Interventional Radiology, Kokilaben Dhirubhai Ambani Hospital and Research Centre, Mumbai, India

**Keywords:** Anterior cruciate ligament, cyclops lesion, localized anterior arthrofibrosis, MRI

## Abstract

Localized anterior arthrofibrosis (cyclops lesion) is the second most common cause of extension loss after anterior cruciate ligament (ACL) reconstruction. We present and discuss two patients with prior ACL reconstructions, who presented with pain and loss of extension following surgery. MRI and arthroscopy of the knee revealed typical features of a cyclops lesion. The patients showed significant symptomatic improvement following arthroscopic resection of these lesions.

## Introduction

Cyclops lesion or localized anterior arthrofibrosis, an arthroscopically treatable complication of anterior cruciate ligament (ACL) reconstruction, is a fibrous nodule located in the intercondylar notch anterior to the ACL graft.[1] The lesion has a typical MRI and arthroscopic appearance. We describe MRI features of the cyclops lesions in two patients who presented with restriction of motion following ACL reconstruction.

## Case Reports

### Case 1

**Figure 1:**
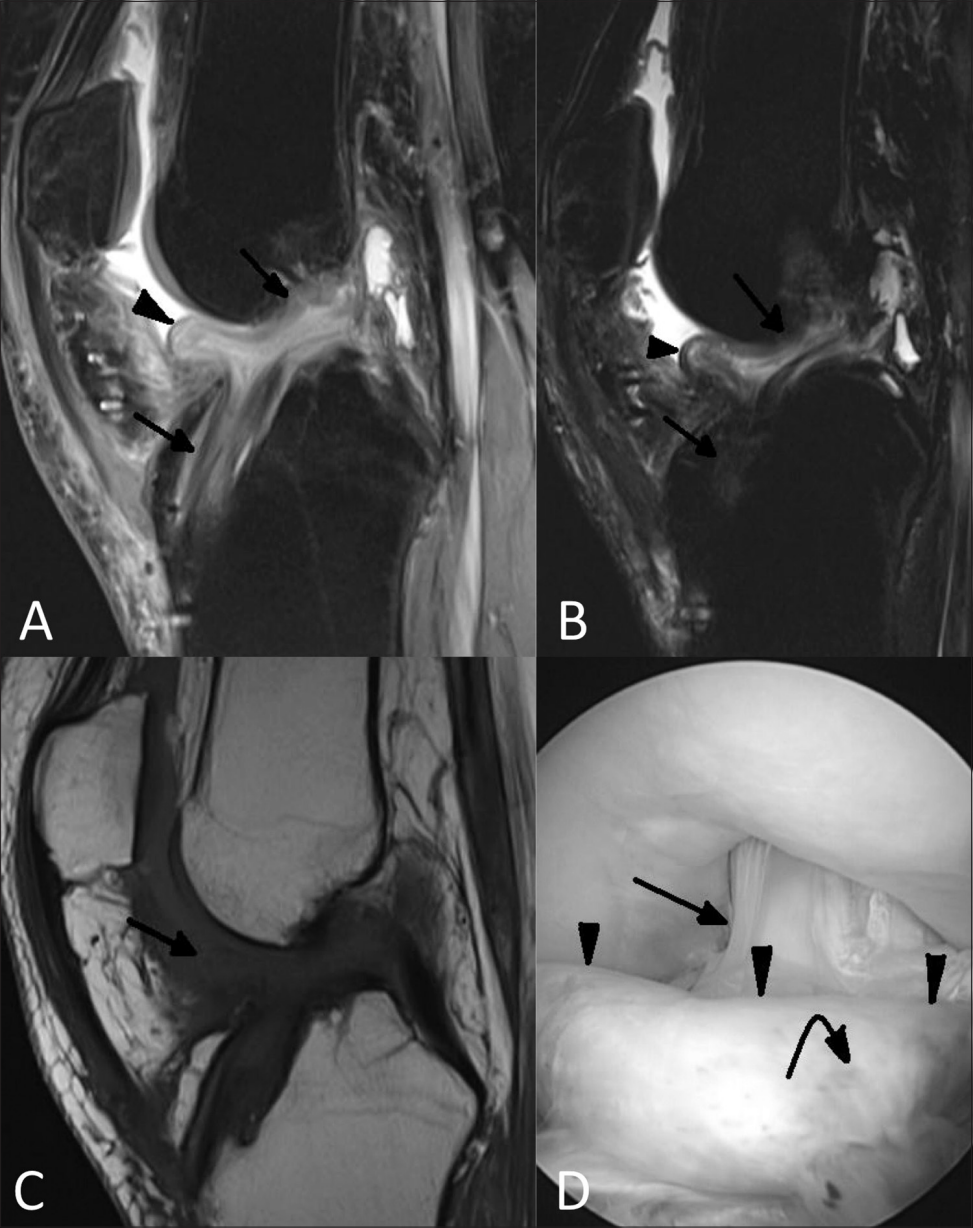
(A-D) Case 1: Fat-suppressed sagittal proton-density weighted (A) and T2W (B) MRI images show an anterior cruciate ligament (ACL) graft (arrow) with a hypointense nodule (arrowhead) attached to its anterior surface in the intercondylar notch. Sagittal T1W MRI image (C) shows the hypointense nodule (arrow) to be indistinguishable from the synovial fluid. Arthroscopic image (D) shows the cyclops lesion (arrowheads) attached to the ACL (arrow) with a head-like appearance, showing a focal area of discoloration resembling an eye (curved arrow)

An 18-year-old boy had undergone left ACL reconstruction 8 months back. He presented with pain and stiffness of the left knee for the last 6 months. MRI [Figure 1A–C] was performed to diagnose the cause of stiffness and also to evaluate meniscal and chondral integrity, tunnel size and position. A soft tissue nodule was seen in the intercondylar notch anterior to and attached to the reconstructed ACL. The nodule appeared hypointense on T1W and isointense to muscle on T2W and proton density-weighted (PDW) images. The tunnel position and size were adequate. Both the menisci appeared normal in shape, configuration and signal intensity. The cartilage lining the tibial, femoral and patellar articular surfaces appeared normal in thickness and signal intensity. On arthroscopy, the nodule had a head-like appearance with a focal area of discoloration resembling an eye [Figure 1D]. In view of the typical clinical, radiological and arthroscopic features, the diagnosis of a cyclops lesion was made. The lesion was treated with arthroscopic excision. Histopathology examination of the specimen showed fibrocartilagenous tissue. Follow-up after 1 month showed full extension of the knee joint.

### Case 2

**Figure 2:**
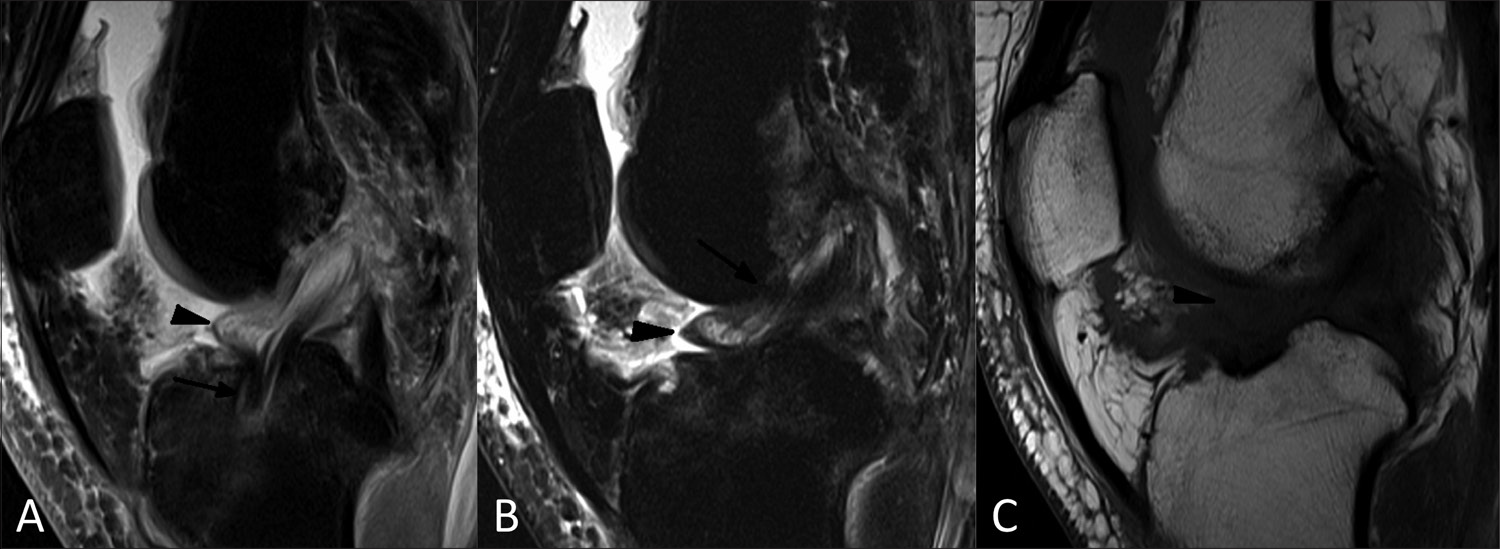
(A-C) Case 2: Fat-suppressed sagittal proton-density weighted (A) and T2W (B) MRI images show a hypointense to isointense nodule (arrowhead) attached to the anterior surface of the anterior cruciate ligament (ACL) graft (arrow). Sagittal T1W MRI image (C) shows a hypointense nodule (arrowhead) in the anterior intercondylar notch

A 52-year-old man presented with pain and extension loss of the left knee for 3 months after ACL reconstruction surgery performed 6 months back. MRI [Figure 2A–C] revealed a soft tissue nodule in the anterior intercondylar notch contiguous with and attached to the reconstructed ACL. The nodule appeared hypointense on T1W and isointense to hypointense on T2W and PDW images. The lesion had a bulbous head-like appearance with a characteristic focal area of reddish-blue discoloration on arthroscopy. These clinical, radiological and arthroscopic features favored the diagnosis of a cyclops lesion. The nodule was arthroscopically excised and routine post-operative mobilization was prescribed. On histopathology, the nodule showed central granulation tissue surrounded by dense fibrous tissue. Significant improvement in the range of extension was noted during the post-operative period.

## Discussion

MRI is the primary post-operative investigative tool in patients with failed ACL reconstruction and is used to evaluate complications, the cause of graft failure, post-operative re-injury and pre-operative planning for repeat surgery. Important complications of ACL reconstruction include graft instability, disruption, extension loss, hardware failure (screw displacement and bone plug dislodgment) and patellar fracture (unique to the use of the patellar tendon autograft).[1]

Loss of motion, particularly loss of knee extension, is a frequent cause of morbidity in patients with prior ACL reconstructions. Localized anterior arthrofibrosis, also called the cyclops lesion, is the second most common cause of extension loss after ACL reconstruction, with a frequency of 1–9.8%, the most common being graft impingement, which develops due to anterior placement of the tibial tunnel.[2] Other less-frequent causes of loss of extension include suprapatellar or intercondylar adhesions, fibrosis of the fat pad, entrapment of the patella and capsular contracture.[2] The pathogenesis of the cyclops lesion is multifactorial; it may be due to debris raised during drilling of the tibial tunnel or impingement of the exposed fibers of the ACL on the intercondylar notch.[2–4]

On histopathology, the lesion consists of central granulation tissue surrounded by dense fibrous tissue.[4] The cyclops lesion has been shown to evolve from an early stage showing fibrosis to a late stage showing fibrocartilaginous soft tissue. Fibrous tissue, fibrocartilage, bone, synovium and fat from the infrapatellar fat pad may all contribute towards the formation of the cyclops lesions.[5]

The nodule is located in the intercondylar notch anterior to the ACL graft. It may be attached to the graft fibers via a pedicle. The nodule gets pinched between the tibia and femur, which then causes a mechanical block to terminal extension. On MRI, the lesion has signal characteristics consistent with fibrous tissue. On T1W images, it may be indistinguishable from the adjacent joint fluid due to its low signal intensity. However, on T2W images, it has a heterogenous low signal intensity, enabling clear differentiation from high signal intensity joint fluid. On MRI arthrography, it can be outlined against the intra-articular contrast.[3] On arthroscopy, the lesion has a head-like appearance with a focal area of reddish-blue discoloration due to venous channels that resemble an eye. Hence, it is called the “cyclops lesion.” The lesion is removed arthroscopically with additional notchoplasty if necessary.[2,3,6] Aggressive physical therapy does not improve extension loss associated with cyclops lesions.[7,8]

The cyclops syndrome was first described by Jackson and Schaefer in patients with ACL reconstruction as a condition presenting with loss of full extension, with development of an audible and palpable “clunk” in terminal extension.[6] The cyclops syndrome has also recently been reported in patients with ACL injury without a history of reconstructive surgery.[6] Additionally, cyclops nodules have also been described in post-trauma patients with a clinically or radiologically intact ACL, probably a reaction to microtrauma leading to subclinically torn ACL fibers.[6]

In conclusion, it is important to recognize a cyclops nodule as a possible cause of extension loss in any patient with ACL injury because it is readily amenable to arthroscopic resection and good patient outcome.
